# Metabolic Rewiring Is Essential for AML Cell Survival to Overcome Autophagy Inhibition by Loss of ATG3

**DOI:** 10.3390/cancers13236142

**Published:** 2021-12-06

**Authors:** Fatima Baker, Ibrahim H. Polat, Khalil Abou-El-Ardat, Islam Alshamleh, Marlyn Thoelken, Daniel Hymon, Andrea Gubas, Sebastian E. Koschade, Jonas B. Vischedyk, Manuel Kaulich, Harald Schwalbe, Shabnam Shaid, Christian H. Brandts

**Affiliations:** 1 Department of Medicine II, Hematology/Oncology, Goethe University, 60590 Frankfurt am Main, Germany; baker@med.uni-frankfurt.de (F.B.); polat@med.uni-frankfurt.de (I.H.P.); khalilard@gmail.com (K.A.-E.-A.); thoelken@med.uni-frankfurt.de (M.T.); koschade@med.uni-frankfurt.de (S.E.K.); vischedyk@med.uni-frankfurt.de (J.B.V.); 2 German Cancer Consortium (DKTK), German Cancer Research Center (DKFZ), 69120 Heidelberg, Germany; alshamleh@nmr.uni-frankfurt.de (I.A.); hymon@nmr.uni-frankfurt.de (D.H.); schwalbe@nmr.uni-frankfurt.de (H.S.); 3 Frankfurt Cancer Institute, 60596 Frankfurt am Main, Germany; kaulich@em.uni-frankfurt.de; 4 Center for Biomolecular Magnetic Resonance (BMRZ), Institute of Organic Chemistry and Chemical Biology, Goethe-University, 60438 Frankfurt am Main, Germany; 5 Institute of Biochemistry II, Faculty of Medicine, Goethe University, 60590 Frankfurt am Main, Germany; a.gubas@em.uni-frankfurt.de; 6 Cardio-Pulmonary Institute, 60590 Frankfurt am Main, Germany; 7 University Cancer Center Frankfurt (UCT), University Hospital, Goethe University, 60590 Frankfurt am Main, Germany

**Keywords:** autophagy, ATG3, autophagy inhibition, acute myeloid leukemia, metabolic rewiring

## Abstract

**Simple Summary:**

The importance of autophagy in leukemia progression and survival has been studied previously. However, little is known about the development of resistance mechanisms to autophagy inhibition in leukemia. Here, we present data on the mechanisms by which leukemia cells maintain their cell survival after inhibition of autophagy by the loss of ATG3. After the loss of ATG3, leukemia cells upregulated their energy metabolism by increasing glycolysis and mitochondrial metabolism, in particular oxidative phosphorylation, which resulted in higher ATP levels. Moreover, inhibition of mitochondrial function strongly impaired cell survival in ATG3 deficiency, thus demonstrating the importance of ATG3 in the regulation of metabolism and survival of leukemic cells. Therefore, our data provide a rationale for combining autophagy inhibitors with inhibitors targeting mitochondrial metabolism for the development of leukemia therapy to overcome the potential obstacle of emerging resistance to autophagy inhibition.

**Abstract:**

Autophagy is an important survival mechanism that allows recycling of nutrients and removal of damaged organelles and has been shown to contribute to the proliferation of acute myeloid leukemia (AML) cells. However, little is known about the mechanism by which autophagy- dependent AML cells can overcome dysfunctional autophagy. In our study we identified autophagy related protein 3 (ATG3) as a crucial autophagy gene for AML cell proliferation by conducting a CRISPR/Cas9 dropout screen with a library targeting around 200 autophagy-related genes. shRNA-mediated loss of ATG3 impaired autophagy function in AML cells and increased their mitochondrial activity and energy metabolism, as shown by elevated mitochondrial ROS generation and mitochondrial respiration. Using tracer-based NMR metabolomics analysis we further demonstrate that the loss of ATG3 resulted in an upregulation of glycolysis, lactate production, and oxidative phosphorylation. Additionally, loss of ATG3 strongly sensitized AML cells to the inhibition of mitochondrial metabolism. These findings highlight the metabolic vulnerabilities that AML cells acquire from autophagy inhibition and support further exploration of combination therapies targeting autophagy and mitochondrial metabolism in AML.

## 1. Introduction

Acute myeloid leukemia (AML) develops from malignant clonal expansion of undifferentiated myeloid progenitors, causing bone marrow failure. Despite advances in the treatment of AML, prognosis remains poor for most patients. Consequently, there is an unmet medical need to find novel therapeutic approaches targeting AML onset and progression.

Several studies have underlined the pivotal role of autophagy in the development and progression of AML [1,2]. Autophagy is a well-known regulator of cellular metabolism contributing to homeostasis and cell survival [3]. The self-degradative property of cytosolic macromolecules is central to autophagy thereby serving as a nutrient source for building blocks during limited energy supply [4]. Proliferating cancer cells are exposed to permanent nutrient deprivation and maintain high energy levels by rewiring metabolic pathways [5,6]. In particular, AML cells are highly dependent on mitochondrial metabolism, as we and others have demonstrated the fundamental role of mitophagy in maintaining mitochondrial integrity in AML [7,8,9]. Therefore, numerous studies have presented the promising approach of targeting autophagy together with different chemotherapies for cancer therapy [10]. However, inherent and acquired resistance to autophagy inhibition has been reported in clinical trials, resulting in further tumor progression after initial response [11]. Importantly, autophagy-related proteins (ATG) including ATG5 and ATG7, which are well known critical autophagy genes, have been reported to promote cell proliferation in leukemia [12,13]. Furthermore, the core autophagy gene ATG3, which directly participates in autophagosome formation by conjugating phosphatidyl-ethanolamine with LC3 [14,15,16], was shown to be essential for leukemogenesis [17].

Despite the vast number of studies exploring autophagy, the mechanisms involved in overcoming autophagy inhibition are poorly understood. In this study, we identified ATG3 as a key player for survival of leukemia cells and present conclusive evidence supporting that upon loss of ATG3 AML cells rewire their central carbon metabolism to evade survival disruption upon autophagy inhibition. Firstly, we performed a CRISPR/Cas9 screen using an autophagy library to identify genes essential for AML proliferation. We found ATG3 to be important for AML cell proliferation and essential for autophagy. By analyzing the effect of ATG3 deficiency in AML cell lines, we found increased levels of mitochondrial reactive oxygen species and upregulated oxidative phosphorylation. By utilizing uniformly labeled ^13^C-glucose nuclear magnetic resonance (NMR) analysis, we observed increased glycolysis and enhanced activity of mitochondrial metabolism upon ATG3 loss. Furthermore, ATG3 deficiency sensitized AML cells to inhibition of mitochondrial respiration. These results suggest a combination of inhibitors targeting autophagy and mitochondrial metabolism as promising approach to treat AML.

## 2. Materials and Methods

### 2.1. Cell Culture

Human AML cell lines THP-1, MV4-11, Molm13, HL-60, and HEL276 (purchased from Leibniz-Institut DSMZ-Deutsche Sammlung von Mikroorganismen und Zellkulturen GmbH, Braunschweig, Germany) were grown in RPMI 1640 medium (Gibco, Thermo Fisher Scientific) supplemented with 10% fetal bovine serum (FBS) (Sigma-Aldrich), 2 mM glutamine (Gibco, Thermo Fisher Scientific, Rockford, IL, USA), and 1% penicillin/streptomycin (Gibco, Thermo Fisher Scientific, Rockford, IL, USA). HEK293T cells (purchased from DSMZ, Braunschweig, Germany) were grown in DMEM medium (Gibco, Thermo Fisher Scientific) supplemented with 10% FBS, and 1% penicillin/streptomycin. All cell lines were cultured at 37 °C with humidified and 5% CO_2_ atmosphere in a Heracell 150i incubator (Thermo Fisher Scientific). All cell lines were determined negative for *Mycoplasma* before starting the experiments using the Mycoplasma PCR Detection Kit (Macherey Nagel, Dueren, Germany). Details of the stable knockdown generation, and cell growth assays can be found in the supplementary methods.

### 2.2. CRISPR/Cas9 Proliferation Screen

Cas9-expressing THP-1 and MV4-11 cells were transduced with an autophagy library [18] virus at a low multiplicity of infection (~0.3). Two days after transduction, cells were selected with 2 μg/mL puromycin for 34 days. Cell samples were frozen before puromycin treatment and at day 34 for genomic DNA isolation. Further details are provided in supplementary methods.

### 2.3. Flow Cytometry Analyses

Apoptosis, cell cycle distribution, mitochondrial superoxide levels, mitochondrial mass, mitochondrial membrane potential, autophagy and mitophagy fluxes were measured using the flow cytometer BD LSRFortessa (BD Biosciences, Heidelberg, Germany). Results were analyzed by using Flow Jo software (v10, Ashland, OR, USA). Details can be found in the supplementary methods (File S1).

### 2.4. Immunofluorescence Staining

Autophagosome formation was analyzed by using confocal microscopy imaging as explained in detail in supplementary methods. LC3 punctae were quantified by using ImageJ (NIH).

### 2.5. Assessment of Mitochondrial Activity

To analyze cellular ATP levels, CellTiter-Glo^®^ Luminescent Cell Viability Assay (Promega, Walldorf, Germany, G7571) was performed. The luminescence signal was then normalized to the cell number in each well.

Oxygen consumption rate (OCR) and extracellular acidification rate (ECAR) of cells were measured in real time by the Seahorse XFe96 Analyzer (Agilent). Details can be found in the supplementary methods (File S1).

### 2.6. NMR-Based Metabolomics and Spectrophotometry

Concentrations of different metabolites and incorporation of ^13^C glucose into central carbon metabolism in AML cells upon ATG3 loss were measured by nuclear magnetic resonance (NMR) spectroscopy as described previously [19,20,21]. Extracellular glucose, glutamine, and lactate concentrations were measured using spectrophotometry. Details can be found in the supplementary methods (File S1).

### 2.7. Statistical Analysis

Each experiment (except colony formation assay and NMR analysis) was performed at least 3 times and collated results are represented in the figures. Data are shown as mean ± standard error of the mean (SEM). Using GraphPad Prism software (v9, San Diego, CA, USA), two-tailed paired Student’s *t*-test was performed. Statistical significance was assumed if a null hypothesis could be rejected at p < 0.05. One asterisk (*) denotes *p*-value < 0.05, two asterisks (**) denote *p*-value < 0.01, three asterisks (***) denote *p*-value < 0.001, and four asterisks (****) denote *p*-value < 0.0001.

## 3. Results

### 3.1. Identification of Critical Autophagy Genes for AML Survival by CRISPR/Cas9 Knockout Screen

**Figure 1:**
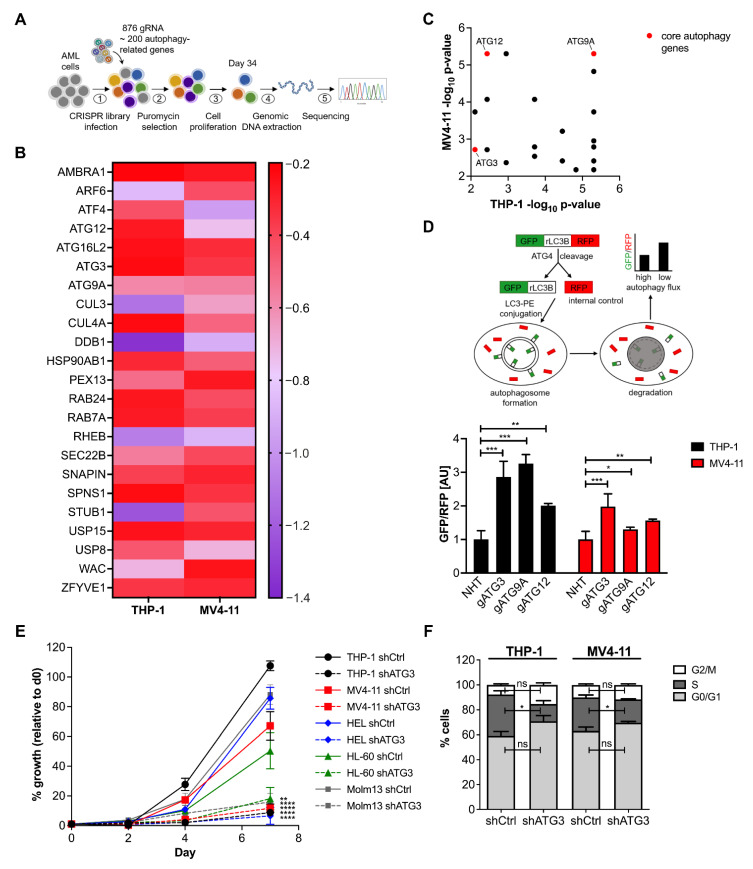
ATG3 drops out in CRISPR/Cas9 screen and loss of ATG3 impairs AML cell proliferation and autophagy. (**A**) Experimental scheme of CRISPR/Cas9 proliferation screen. (**B**) Heat map represents the log_2_ fold change (scaled with indicated colors) of the 23 common dropout genes from the CRISPR/Cas9 screen using the autophagy library in THP-1 and MV4-11 cells. Dropout genes were defined with a log_2_ fold change ≤ −0.2. (**C**) Scatter plot of the common dropout genes according to significance. Significant dropout genes were defined with a −log_10_ *p*-value ≥ 2. (**D**) Autophagy flux was analyzed under steady state conditions using the GFP-LC3B-RFP reporter with flow cytometry in THP-1 cells and MV4-11 cells with CRISPR/Cas9-mediated gene knockout of indicated genes after three days of puromycin selection. (**E**) Cell growth analysis of scrambled nucleotide control short hairpin RNA (shCtrl) and shRNA against ATG3 (shATG3) in THP-1, MV4-11, HEL276, HL-60, and Molm13 cell lines. A total of 1 × 10^4^ cells were seeded at day 0 and counted by trypan blue exclusion at indicated time points. Cell numbers were normalized to d0 and are shown as percentage. (**F**) Cell cycle analysis by flow cytometry of THP-1 and MV4-11 cells stained with BrdU and 7AAD. Bar graphs show quantification of cells in each cell-cycle phase: G0/G1 phase (BrdU negative; 2N DNA content), S-phase (BrdU positive), G2/M phase (BrdU negative; 4N DNA content). Student’s t test was performed in (**D**–**F**). Error bars represent SEM. ns, not significant, * *p* < 0.05, ** *p* < 0.01, *** *p* < 0.001, **** *p* < 0.0001.

To identify critical autophagy genes for AML cell proliferation we performed a CRISPR/Cas9 knockout screen in the two human AML cell lines THP-1 and MV4-11 (schematized in Figure 1A). A library targeting 192 autophagy-related genes including core autophagy genes, autophagy receptors, autophagy regulating transcription factors, and ubiquitin-specific proteases [22] was employed. The library contained four different single guide RNAs (sgRNA) per gene, 876 sgRNAs in total including controls. To maintain a 1000-fold library coverage, 3 × 10^6^ cells were transduced at ~0.3 multiplicity of infection (MOI). Two days after transduction, one sample was harvested as day 0 and the remaining cells were cultivated for 34 days under selection.

A MAGeCK analysis was applied to the screen data sets of both AML cell lines. Sequencing analysis revealed that around 90% of sgRNAs were detectable and sgRNA distribution was equal. We found 74 genes significantly depleted in THP-1 cells and 44 genes were essential in MV4-11 cells with a −log_10_ *p*-value ≥ 2 and a log_2_ fold change ≤ −0.2 (Figure S1A, Table S1). As expected, known essential genes for cell proliferation were among the most depleted genes discovered in our screen (Figure S1A), confirming the sgRNA depletion due to functional genetic knockout phenotype. Overall, the CRISPR mediated viability screen identified 23 genes that were significantly depleted in both cell lines (Figure 1B,C). Notably, similar depletion patterns were observed in the integrated database of CRISPR Screens (iCSDB) [23] representing 40 screens across 24 human leukemia cell lines indicating the reliability of our data (Figure S1B).

### 3.2. Loss of ATG3 Effectively Inhibits Autophagy Flux Accompanied by Increased Cell Death While Retaining Cell Survival

As the core autophagy machinery is rarely mutated in cancers including AML [24], indicating its contribution to cancer survival, we focused our effort on the core autophagy genes. Out of the 23 common dropout genes we identified three core autophagy genes, namely ATG3, ATG9A, and ATG12 (Figure 1B) [25]. We further found the isoform ATG16L2 of the autophagy gene ATG16L, which was shown to be dispensable for autophagosome formation and behaving very differently from other canonical autophagy genes [26,27]. As these findings limit the interpretation of the autophagy-dependent role of ATG16L2, this candidate was not included in further analyses.

To select the hit with the strongest effect on autophagy, we measured autophagy flux by using AML cells expressing a GFP-rLC3B-RPF reporter (as described by Kaizuka et al. [28]). This probe is cleaved by ATG4 proteases into equimolar amounts of RFP and GFP-rLC3B, which is incorporated into the autophagosomal membrane and degraded afterwards, whereas RFP remains as internal control in the cytosol. Thus, the ratio of GFP/RFP signal reversely correlates with autophagic activity and depicts the dynamic of the autophagy process quantitatively in real time without the necessity of using autophagy inhibitors. Among the three analyzed genes, *ATG3* knockout showed the strongest and most consistent autophagy block in THP-1 and MV4-11 cells (Figure 1D). Starvation did not result in an induction of autophagy and bafilomycin treatment for inhibiting autophagy flux did not further block autophagy upon loss of ATG3 (Figure S2A). The autophagy block was in line with LC3 western blot showing reduced LC3 lipidation after the knockdown of ATG3 in AML cells (Figure S2B,C). ATG3 deficiency did not alter the expression of ATG7 and ATG4 in THP-1 and MV4-11 cells (Figure S2D,E), which is in line with previous studies showing no changes of the expression of different core autophagy genes [29,30]. In addition, we assessed autophagosome formation by quantitative immunofluorescence microscopy and observed a decreased number of LC3 punctae in THP-1 and MV4-11 cells upon ATG3 knockdown under untreated conditions and after blocking lysosomal degradation by Baf treatment (Figure S2F,G).

Next, we confirmed that ATG3 is required for unperturbed AML cell growth. We performed proliferation assays in five different human AML cell lines as AML is a very heterogenous disease with several driver-mutations. As AML is a very heterogenous disease with several driver-mutations. For example, FLT3-ITD mutations occur in about 25% of AML and result in a poor prognosis and high frequency of relapse [31] and *TP53* mutations have been associated with poor response to chemotherapy and low overall survival [32]. Therefore, we chose established cell lines that provide a model system to study leukemia proliferation in vitro by using FLT3-ITD positive (MV4-11 [33], Molm13 [34]) and FLT3-wild type (THP-1 [34], HEL [35], HL-60 [33]), as well as p53-wt (MV4-11 [36], Molm13 [37]) and p53-mutated (THP-1 [38], HL-60 [37], HEL [37]) AML cell lines to analyze the loss of ATG3 on AML proliferation. We generated sublines of these cell lines after the knockdown of ATG3 by short hairpin RNA (shRNA). The loss of ATG3 significantly decreased proliferation of all AML cell lines (Figure 1E). This was also reflected in a reduced colony formation capacity in THP-1 and MV4-11 cells upon ATG3 knockdown (Figure S3A). Interestingly, we observed that all AML cell lines were still able to proliferate upon loss of ATG3 (Figure 1E), as cell cycle analysis in THP-1 and MV4-11 cells revealed a reduced S-phase without arresting the cell cycle (Figure 1F and Figure S3B). Although we detected increased apoptosis in both cell lines after depletion of ATG3, 45–50% of cells were still alive (Figure S3C,D). These findings strongly suggest that leukemia cells are able to overcome autophagy inhibition by the loss of ATG3.

### 3.3. Mitochondrial Homeostasis Maintained by Mitophagy Is Not Disrupted by Inhibition of Autophagy upon Loss of ATG3

**Figure 2:**
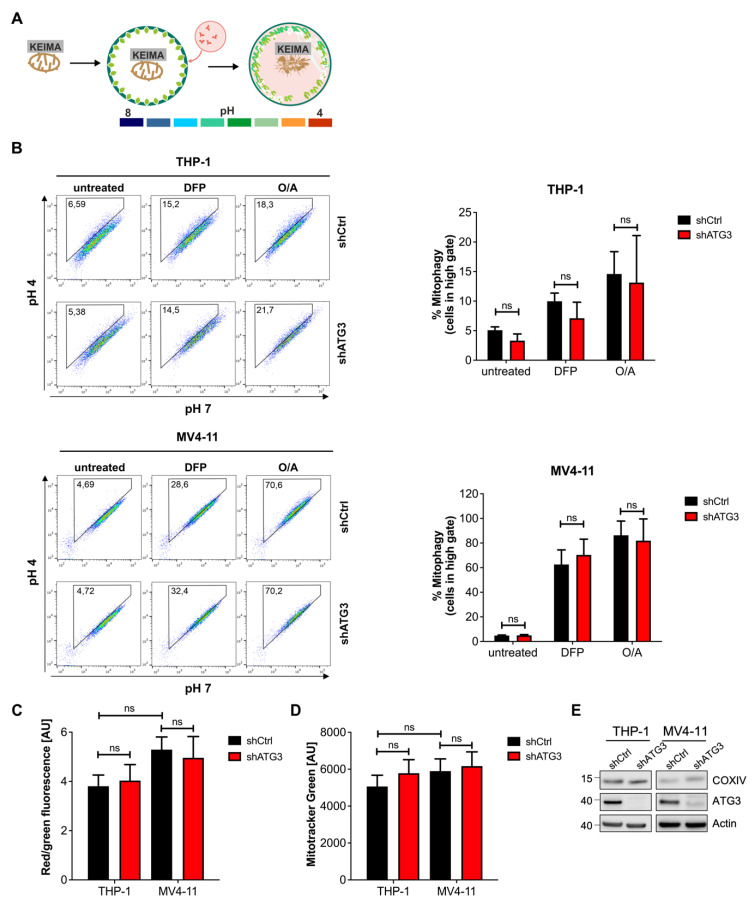
Loss of ATG3 does not affect mitochondrial homeostasis. (**A**) Scheme of the mitophagy reporter mt-mKEIMA. (**B**) Mitophagy was assessed using mt-mKEIMA expressing cells. Cells were treated with 400 µM deferiprone (DFP) for 16 h, 10 µM oligomycin and 10 µM antimycin A (O/A) for 8 h, or vehicle control, and analyzed by flow cytometry. A shift of the cell population into the gate depicts mitophagy induction. Representative dot plots are shown. Quantification of mitophagy was determined by the percentage of cells shifting into the gate. (**C**) Analysis of mitochondrial membrane potential by JC-1 staining and flow cytometry measurements. The quantification of the ratio of MFI Red and MFI Green is shown with red indicating intact mitochondria and green indicating depolarized mitochondria. (**D**) Mitochondrial mass was analyzed by MitoTracker Green staining and measured by flow cytometry. (**E**) Representative western blot image showing the expression of the mitochondrial protein COXIV upon ATG3 depletion by shRNA. Student’s t test was performed in (**B**–**D**). Error bars represent SEM. ns, not significant.

As our data demonstrated that leukemia cells can survive the inhibition of ATG3-dependent autophagy, we wanted to elucidate the mechanism contributing to maintaining cellular homeostasis and survival. We and others have previously reported that in AML degradation of damaged mitochondria by mitophagy is critical for sustaining mitochondrial homeostasis and cell survival [2,7,8]. Therefore, we applied a quantitative and sensitive mitophagy assay by using cells expressing mitochondrial targeted mt-mKEIMA reporter [39] (Figure 2A). This fluorescent protein reversibly changes its color dependent on neutral or acidic pH and can be used to monitor the conversion of autophagosomes to autolysosomes by fusion with acidic lysosomes. To trigger mitophagy, we induced mitochondrial damage in two different ways: (i) By inhibiting the ATPase and complex III of the mitochondrial respiratory chain with oligomycin and antimycin A (O/A) respectively, mitochondrial depolarization was induced; and (ii) the iron chelator deferiprone (DFP) causes intracellular iron depletion thereby disturbing mitochondrial iron homeostasis, which results in mitophagy induction. Interestingly, a substantial fluorescent shift was observed after 8 h of O/A treatment and 16 h of DFP in THP-1 and MV4-11 cells, which was independent of ATG3 expression (Figure 2B). In line with this, loss of ATG3 did not affect mitochondrial polarization, as JC-1 staining followed by flow cytometry analysis revealed no difference on red/green fluorescence ratio of control and knockdown THP-1 and MV4-11 cells (Figure 2C and Figure S4A). Furthermore, we determined total mitochondrial mass in THP-1 and MV4-11 cells by mitotracker green staining and flow cytometry analysis. As shown in Figure 2D and Figure S4B, the presence or absence of ATG3 did not affect overall mitochondrial mass. Additionally, western blot analysis of the mitochondrial protein COXIV did not show different expression levels in control and ATG3-depleted cells (Figure 2E and Figure S4C). We conclude that the loss of ATG3 does not affect mitochondrial homeostasis as neither mitophagy nor mitochondrial mass was altered.

### 3.4. Loss of ATG3 Increases Mitochondrial Activity

**Figure 3:**
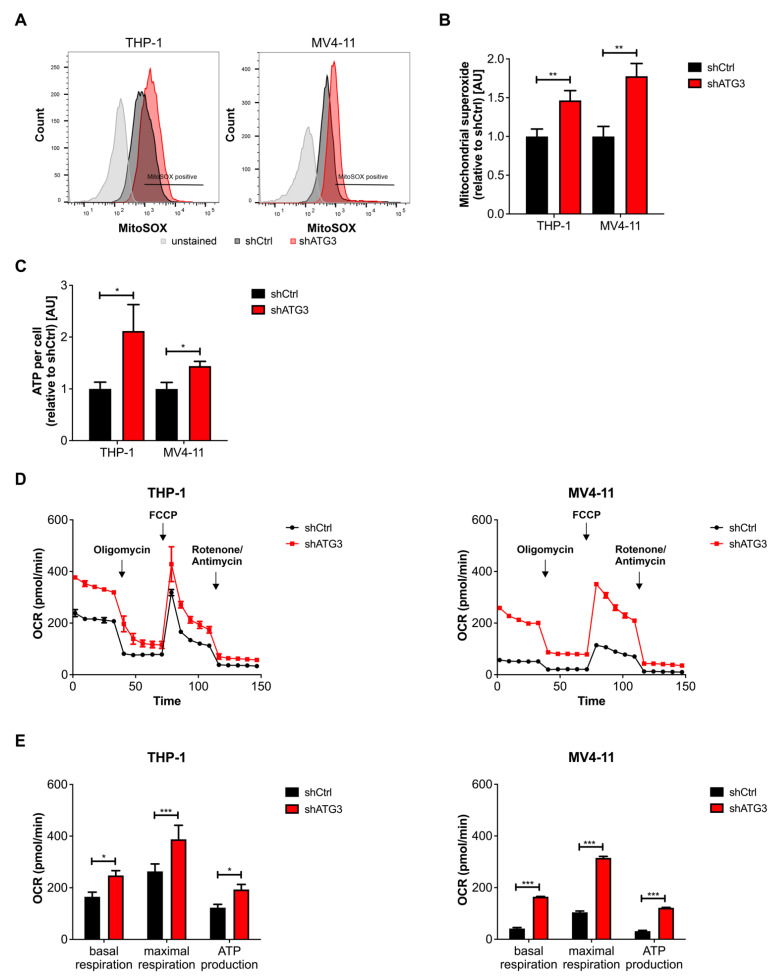
Loss of ATG3 increases mitochondrial activity in AML cells. (**A**) Mitochondrial superoxide levels were determined in THP-1 and MV4-11 cell lines by flow cytometry using MitoSOX dye. Representative flow cytometry images are shown. (**B**) Quantification of mitochondrial superoxide levels. (**C**) ATP levels of control and ATG3 knockdown cells were determined using Cell Titer Glo^®^ luminescent cell viability assay and normalized to cell number counted by trypan blue exclusion after 72 h of incubation. Bar graph depicts quantification of ATP levels normalized to control. (**D**) Mitochondrial respiration of control and ATG3 knockdown cells was measured by oxygen consumption rate (OCR) in real time by the Agilent Seahorse XFe96 Analyzer. Representative curves are shown. (**E**) Quantification of basal respiration, maximal respiration, and ATP production were calculated from OCR measurements. Student’s t test was performed in (**B**,**C**,**E**). Error bars represent SEM. * *p* < 0.05, ** *p* < 0.01, *** *p* < 0.001.

Under the assumption that mitochondrial turnover was not affected, we hypothesized that the loss of ATG3 contributes to mitochondrial function to sustain cell survival. Taking into account that reactive oxygen species (ROS) are generated as by-products during oxidative phosphorylation (OXPHOS) within mitochondria [40], we assessed mitochondrial fitness by measuring mitochondrial superoxide (MitoSOX). Indeed, MitoSOX staining with flow cytometric analysis revealed increased levels of mitochondrial ROS upon ATG3 depletion in THP-1 and MV4-11 cells (Figure 3A,B). In line with this, we also detected increased ATP levels using luminescence viability assays in THP-1 and MV4-11 cells with ATG3 knockdown (Figure 3C). Furthermore, to accurately quantify mitochondrial activity, we measured mitochondrial OXPHOS based on the oxygen consumption rate (OCR) through real-time and live cell analysis by using Seahorse XFe96 Flux analyzer. Importantly, our detailed analyses revealed increased OCR in both THP-1 and MV4-11 cells after ATG3 knockdown compared to control cells (Figure 3D). For quantifying mitochondrial respiration, we analyzed basal respiration, which represents the energetic demand of cells under baseline conditions, and the maximum rate of respiration that can be achieved by the cells. We observed increased basal and maximal respiration in both cell lines upon ATG3 knockdown (Figure 3E). We also quantified ATP production by mitochondria from the OCR measurements upon oligomycin injection and found increased ATP production in ATG3 knockdown cells, which is in line with our previous measurement (Figure 3E). Furthermore, proton leak and non-mitochondrial respiration were modestly increased upon loss of ATG3 (Figure S5). However, the rate of oxygen consumption for non-mitochondrial respiration and proton leak was very low compared to basal respiration and ATP-linked respiration, respectively. Taken together, our data demonstrate that loss of ATG3 upregulates mitochondrial activity by oxidative phosphorylation in AML cells.

### 3.5. Loss of ATG3 Rewires Central Carbon Metabolism

**Figure 4:**
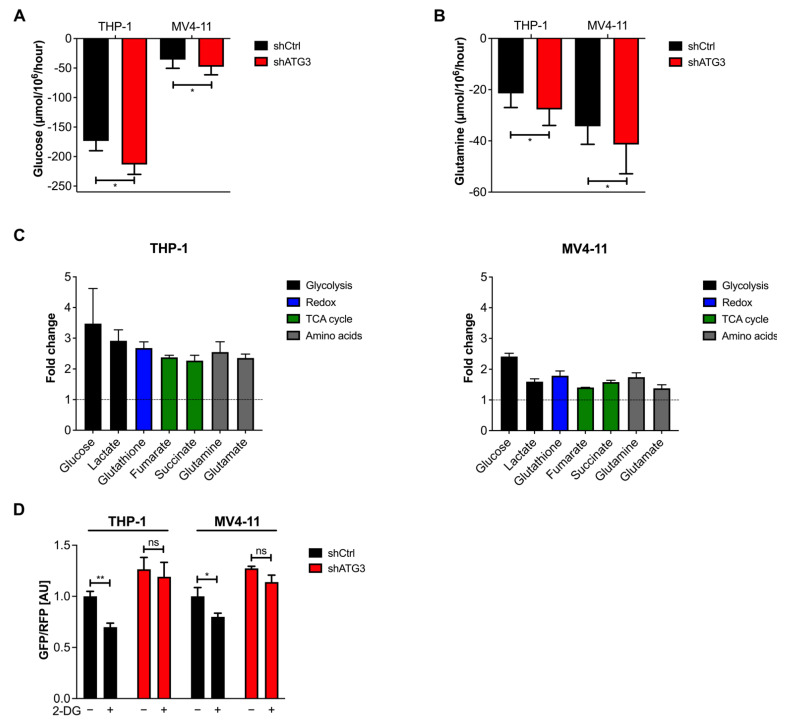
AML cells rewire their central carbon metabolism upon loss of ATG3. (**A**) Glucose and (**B**) glutamine consumption fluxes are shown. Control or shATG3 cells were incubated for 72 h, and medium was collected afterwards. Glucose and glutamine fluxes were calculated by measuring extracellular glucose and glutamine concentrations by spectrophotometric assays and normalizing it to the cell number. (**C**) Intracellular concentrations of central metabolites depicted as fold change (normalized to control) measured by NMR. (**D**) Autophagy flux was measured with the GFP-LC3B-RFP reporter in THP-1 and MV4-11 cells and normalized to the shCtrl untreated condition. The 1 mM 2-DG treatment was performed for 24 h. Student’s t test was performed in (**A**,**B**,**D**). Error bars represent SEM. ns, not significant, * *p* < 0.05, ** *p* < 0.01.

To understand the effects by which ATG3 loss leads to increased oxidative phosphorylation and increased mitochondrial superoxide production, we analyzed the metabolic alterations in AML cells. We first analyzed the glucose and glutamine consumption of THP-1 and MV4-11 cells after ATG3 knockdown. To this end, we first measured the extracellular concentration of these two metabolites by spectrophotometry after 72 h and calculated their influx in both AML cell lines. We observed that ATG3 depletion increased the glucose and glutamine consumption in THP-1 and MV4-11 cells (Figure 4A,B). To identify the metabolic changes upon autophagy inhibition in greater detail, we performed nuclear magnetic resonance (NMR) spectroscopy measurements to determine the intracellular concentrations of key metabolites in THP-1 and MV4-11 cells in the absence of ATG3. We observed increased intracellular concentrations of glutathione, which is an antioxidant used for redox balance. Moreover, intracellular concentrations of the key molecules of central carbon metabolism, such as glucose, lactate, glutamine, and glutamate were increased. Intracellular concentrations of certain tricarboxylic acid (TCA) cycle elements, namely fumarate and succinate, were also increased (Figure 4C). In summary, these data strongly indicate that upon loss of ATG3, AML cells upregulate their energy metabolism.

### 3.6. The Glycolytic Pathway Is Highly Active in AML Cells upon Loss of ATG3

Given the fact that NMR analysis revealed an increased intracellular glucose concentration, which was accompanied by enhanced glucose influx in ATG3-depleted AML cells, we first focused on glucose metabolism. Since glycolysis is reported to regulate autophagy [41], we could confirm that inhibition of glycolysis with the glucose analog 2-deoxy glucose (2-DG) induced autophagy flux in control AML cells, whereas loss of ATG3 blocked autophagy induction (Figure 4D).

**Figure 5:**
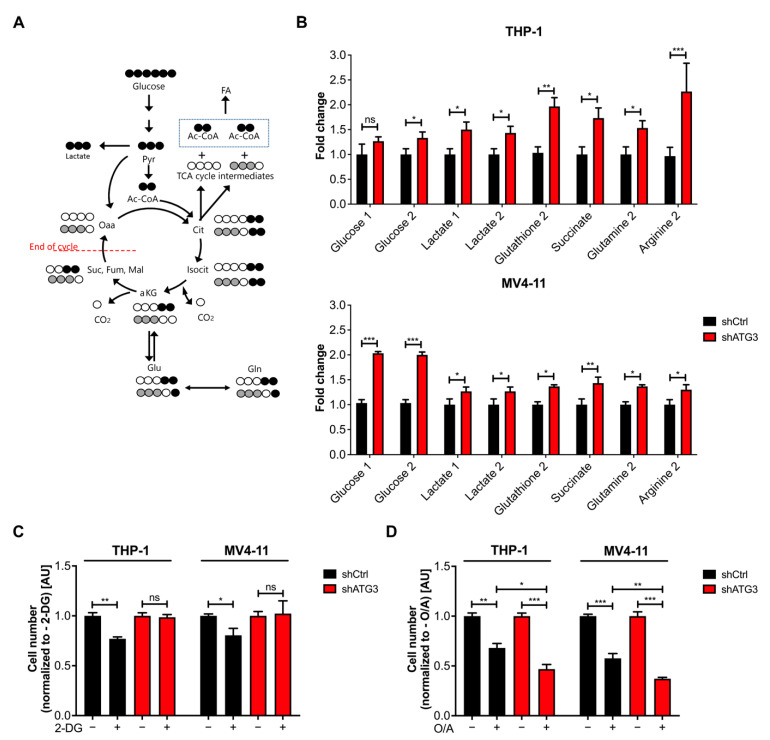
Loss of ATG3 activates glucose metabolism and TCA cycle but sensitizes AML cells to mitochondrial inhibition. (**A**) Carbon atom transition map when using [U-^13^C_6_]-glucose as a tracer. Pyruvate entry into mitochondria can take place in two ways: via pyruvate dehydrogenase (PDH) or via pyruvate carboxylase (PC). For each metabolite, black molecules demonstrate the ^13^C labeling pattern of Ac-CoA entry into TCA cycle by PDH corresponding to the first cycle of oxidation. On the other hand, gray molecules indicate the pyruvate entry into TCA cycle by PC. For clarity reasons, only the first turn of TCA cycle is depicted here. (**B**) Fold change of indicated metabolites normalized to control (shCtrl) of indicated metabolites analyzed by tracer-based NMR using [U-^13^C_6_]-glucose. (**C**) Cell growth analysis of THP-1 and MV4-11 control and ATG3-depleted cells in the presence or absence of 1 mM 2-DG or (**D**) 2 µM (THP-1) or 1 µM (MV4-11) oligomycin and antimycin A (O/A, 1:1 ratio). Cells were incubated for 24 h with inhibitors and counted afterwards using trypan blue exclusion. Cell numbers were normalized to untreated conditions. Student’s t test was performed in (**B**–**D**). Error bars represent SEM. ns, not significant, * *p* < 0.05, ** *p* < 0.01, *** *p* < 0.001.

To understand the consequence of increased glucose consumption in autophagy deficient AML cells, we followed the fate of carbon derived from glucose in the presence and absence of ATG3. Therefore, THP-1 and MV4-11 cells were cultured in isotope-labelled glucose (U-^13^C_6_-glucose) medium for 24 h, followed by tracer-based NMR measurements (Figure 5A). Significant labelling was observed in polar metabolites of secondary metabolites derived from glycolytic intermediates and end-products. Furthermore, ATG3 knockdown cells displayed an enrichment of ^13^C labelled intracellular glucose, as well as a significantly increased incorporation of ^13^C into lactate (Figure 5B). Interestingly, extracellular lactate levels measured by spectrophotometry showed no increase of extracellular lactate concentration in ATG3-depleted cells although a concomitant accumulation of intracellular lactate was observed (Figure S6A). To confirm this observation, extracellular acidification rate (ECAR) measurements were used to determine the lactate excretion rate. ECAR was detected under basal conditions followed by the sequential addition of glucose, oligomycin, and 2-DG (Figure S6B,C). We found reduced ECAR after glucose injection in ATG3-deficient cells. Inhibition of mitochondrial ATP production with oligomycin showed no significant increase of ECAR in the absence of ATG3. Injection of 2-DG caused a decrease in ECAR in control and ATG3-deficient cell lines, confirming that the ECAR is generated due to glycolysis. Taken together, our data demonstrate that the glycolytic pathway is highly active in AML cells upon loss of ATG3, leading to increased conversion into intracellularly retained lactate.

### 3.7. Survival of ATG3 Deficient AML Cells Relies on OXPHOS

The discovery that glycolysis is highly activated and ATP levels are increased in the absence of ATG3 suggests that metabolic rewiring plays an important role in the survival of these AML cells. As our data demonstrated that the loss of ATG3 causes increased OXPHOS and elevated intracellular concentrations of certain TCA cycle intermediates (Figure 4C), we analyzed the mitochondrial metabolism by following the ^13^C incorporation of our tracer-based NMR experiment. We found a significant enrichment of labelled carbon derived from glucose in the TCA cycle intermediate succinate, demonstrating the activation of mitochondrial metabolism upon ATG3 depletion. In line with the previous results, we also found significant ^13^C incorporation in glutathione upon ATG3 depletion in both cell lines, suggesting that loss of ATG3 upregulates the production of glutathione for maintaining redox homeostasis of AML cells. In addition, glutamine and arginine showed elevated ^13^C incorporation, indicating their production from a-ketoglutarate (Figure 5A,B).

As many cancer cells rely on glutaminolysis [42], which converts glutamine into TCA cycle intermediates via glutamate for cell survival, we proceeded to investigate the dependency on glutamine. THP-1 and MV4-11 cells were cultured in the presence and absence of glutamine. Interestingly, glutamine deprivation caused impaired cell proliferation independently of ATG3 in both AML cell lines (Figure S6D). In order to understand the mechanism contributing to cell survival in ATG3 deficiency we examined the dependency of leukemia cells on glycolysis or OXPHOS. Therefore, THP-1 and MV4-11 cells were treated for 24 h with either the glycolysis inhibitor 2-DG or OXPHOS inhibitor O/A in the presence and absence of ATG3. Proliferation assay revealed that in the presence of ATG3 both AML cell lines were sensitive to 2-DG and O/A treatment (Figure 5C,D). Notably, upon ATG3 loss AML cells were resistant to glycolysis inhibition as they showed no proliferation defect after 2-DG treatment (Figure 5C). Strikingly, the OXPHOS inhibitors oligomycin and antimycin significantly reduced cell growth compared to control cells (Figure 5D). These results suggest that the survival of ATG3-deficient AML cells relies on mitochondrial OXPHOS.

## 4. Discussion

**Figure 6:**
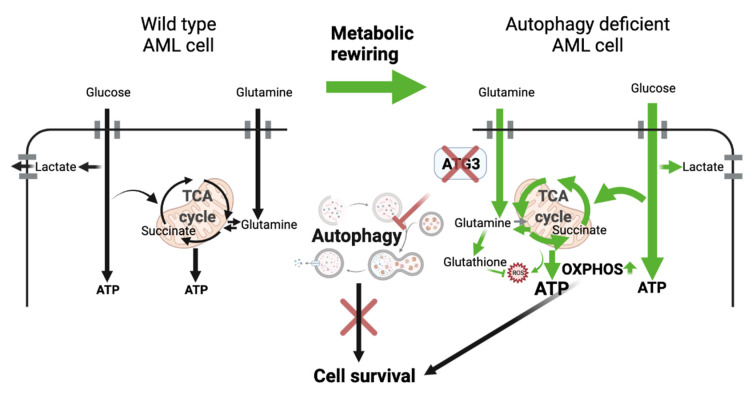
Model of metabolic rewiring in AML cells upon loss of ATG3. Loss of ATG3 upregulates the intracellular glucose uptake and glycolysis thereby increasing lactate production. Lactate export, however, is not increased in ATG3-deficient cells, resulting in an accumulation of lactate within the cells. ATG3 deficiency further results in an upregulation of OXPHOS with elevated ATP levels accompanied by enhanced mitochondrial ROS generation. This metabolic rewiring promotes cell survival in ATG3-deficient cells and increases their dependence on OXPHOS. The model was created with BioRender.com, accessed on 25 November 2021.

In our study, we identified ATG3 as a core autophagy gene essential for AML cell survival by performing a CRISPR/Cas9 proliferation screen targeting 192 autophagy-related genes. By genetically depleting ATG3 in human leukemia cell lines, we demonstrate that AML cells rewire their energy metabolism to sustain cell survival when autophagy is impaired. NMR analysis revealed upregulation of glycolysis and OXPHOS upon ATG3 deficiency, which was accompanied by increased mitochondrial ROS and ATP production. Importantly, while ATG3-deficient cells are resistant to inhibition of glycolysis, we show that inhibition of OXPHOS severely reduces AML cell survival, identifying their dependence on mitochondrial metabolism as arising vulnerability upon autophagy inhibition (Figure 6).

Previous studies have underlined the importance of core autophagy genes for leukemia cell survival; however, there is inconsistency regarding the role of autophagy in modulating leukemia progression, as deletion of certain autophagy genes either enhanced or prevented apoptosis. It was shown that loss of the core autophagy genes ATG5 and ATG7 induced apoptosis and decreased leukemia progression in a murine leukemia model [12]. On the other hand, deletion of *ATG5* and *ATG7* promoted AML cell proliferation [43]. However, this paradoxical role of autophagy depends on the stage of tumorigenesis as it is a pro-survival pathway for established leukemia [44]. Several CRISPR/Cas9 screens with genetic knockout of various core autophagy genes resulted in a strong autophagy inhibition which negatively correlated with proliferation [22,45]. Similarly, among the 23 identified dropout hits from our screen, we found four core autophagy genes to be essential for AML cell survival (Figure 1B,C). Particularly loss of ATG3 was shown to suppress leukemogenesis by promoting apoptosis [17], which is consistent with our data showing reduced proliferation upon knockdown of ATG3 in different AML cell lines harboring different driver mutations (Figure 1E). Reduced proliferation was accompanied by a reduced S-phase of the cell cycle and increased apoptosis in our human AML cell lines (Figure 1F and Figure S3C,D). These results demonstrate the important role of ATG3-mediated autophagy in leukemia cell survival.

The integral role of autophagy in maintaining mitochondrial homeostasis has been shown by several studies as knockout of core autophagy genes including *ATG3* [46,47,48] in cancer cells severely disrupts mitochondrial homeostasis leading to disorganized cristae, reduced oxidative phosphorylation, accumulation of fragmented mitochondria, and impaired mitophagy [49,50]. On the contrary it has been demonstrated that mitophagy mainly occurs through an alternative autophagy pathway independent of ATG genes [51]. Likewise, we observed that mitochondrial degradation by mitophagy was not disturbed in the absence of ATG3 (Figure 2B). Moreover, mitochondrial function and mitochondrial turnover were not altered as mitochondrial membrane potential and mitochondrial mass remained stable upon loss of ATG3 (Figure 2C–E and Figure S4). Hence, to our knowledge this is the first study describing ATG3 as dispensable for maintaining mitochondrial homeostasis by mitophagy. So far, little is known about the role of ATG3 in mitochondrial homeostasis.

Mitochondrial function is essential for stemness, migration, and drug resistance of cancer stem cells [52]. It has been reported that ATG7 deletion caused elevated ROS levels and mitochondrial activity in leukemia cells [12,53]. Similarly, we found upregulated mitochondrial OXPHOS in ATG3-deficient AML cell lines, which likely resulted in the increase of mitochondrial superoxide levels (Figure 3A,B). The increase in basal respiration in ATG3-deficient AML cells might be (partially) explained by increased substrate availability for mitochondrial respiration like ADP or succinate [54], as we found increased levels of succinate upon loss of ATG3 (Figure 4 and Figure 5). However, measurement of mitochondrial respiration by Seahorse analysis limits the assessment of ATP levels as the usage of inhibitors as a tool do not completely reflect the physiological process of mitochondrial respiration [55]. Furthermore, a deeper analysis of mitochondrial membrane potential would be necessary to understand the mechanisms leading to the increase in OXPHOS. As the mitochondrial membrane potential by JC-1 analysis was not impaired upon loss of ATG3, membrane depolarization was not responsible for the increased mitochondrial ROS as reported by Sundqvist et al. [56]. Upregulated OXPHOS was reflected by increased OCR and elevation of the TCA cycle intermediates succinate and fumarate (Figure 3D,E and Figure 4C). Loss of ATG5 and ATG7 was found to increase basal and maximal respiration as well as proton leak in cancer cells [57]. Likewise, we observed increased respiration and proton leak in our AML cell lines upon loss of ATG3 (Figure S5), which could either point towards a damaged electron transport chain and/ or mitochondrial membrane amongst others allowing proton leakage [58], or proton leak may reduce the oxidative damage by decreasing superoxide production [59]. However, mitochondrial membrane potential was not affected, and superoxide levels were increased upon loss of ATG3 (Figure 2C and Figure 3A,B), and the levels of proton leak were low compared to ATP synthesis suggesting that leaking protons play a minor role in ATG3 deficiency. An elevated non-mitochondrial respiration in ATG3-depleted AML cells (Figure S5) is in line with ATG5 and ATG7 deficient urinary bladder cancer cells [57] and suggests that oxygen is partially used by non-mitochondrial sources like peroxisomes and NADPH-oxidases [60,61], which could result in increased cytosolic ROS [58], which we also observed by dihydrorhodamine 123 staining upon loss of ATG3 (data not shown). Furthermore, detection of increased intracellular glutamine concentration and glutathione production supports upregulation of mitochondrial metabolism since glutamine is one of the major energy sources of mitochondria (Figure 4B,C and Figure 5B). On the other hand, autophagy-deficient cancer cells showed decreased ATP levels and reduced nucleotide pools during starvation, which could be rescued by glutamine supplementation thereby feeding the TCA cycle and maintaining mitochondrial function [50]. In proliferating cells glutamine is used as a carbon source for TCA cycle intermediates including succinate and fumarate [62]. Glutamine is also the major precursor of the ROS scavenger glutathione. Elevated glutathione levels are observed in several tumor types including leukemias [63] and are also associated with drug resistance in tumor cells [64]. Notably, glutathione is particularly important in neutralizing peroxides generated in mitochondria [65]. We showed that loss of ATG3 causes an excessive generation of ROS which was accompanied by increased glutathione levels implying that deficiency of ATG3 is compensated by altered redox state contributing to cell survival. Moreover, NADPH is used for ROS detoxification, which is mainly produced at the oxidative branch of the pentose phosphate pathway, but also conversion of malate to pyruvate by malic enzyme and conversion of isocitrate to alpha-ketoglutarate by isocitrate dehydrogenase are significant sources of NADPH [66]. Therefore, increased TCA cycle activity also produces more NADPH, which in turn is required for scavenging ROS.

Metabolic rewiring allows cancer cells to switch between glycolysis and oxidative phosphorylation to adapt to changing conditions during cancer progression [67]. By using tracer-based NMR analysis we found increased glycolysis upon ATG3 loss measured by glucose uptake and consumption as well as lactate production (Figure 4A,C and Figure S6A). Surprisingly, our results demonstrated decreased lactate excretion (Figure S6B,C). In glycolytic cancer cells, lactate is exported to prevent intracellular acidification and subsequent cell death [68]. Therefore, it might be possible that ATG3-dependent autophagy contributes to the regulation of intracellular pH by lactate excretion and that loss of ATG3 impairs this capacity, leading to intracellular lactate accumulation. On the other hand, lactate is used as a fuel source by being oxidized to pyruvate and enters the TCA cycle for energy and amino acid production, and lipid biosynthesis among others [69,70]. Thus, ATG3-depleted AML cells might metabolize the produced lactate intracellularly without exporting it. However, to understand the reduced lactate export rate and the fate of lactate in the cell in ATG3 deficiency needs further investigation.

Numerous studies have shown that autophagy has a profound impact on metabolism [71]. Glucose has been reported to regulate autophagy in different cancers, as inhibition of glycolysis activates autophagy [41,72]. Similarly, we observed increased autophagy flux in THP-1 and MV4-11 AML cells upon inhibition of glycolysis by 2-DG (Figure 4D). Notably, this was accompanied by impaired cell proliferation. However, we noted that ATG3-depleted AML cells were still able to proliferate (Figure 5C). Moreover, we detected increased levels of fumarate and succinate after ATG3 loss in THP-1 and MV4-11 cells indicating a more active TCA cycle, which in turn implies increased OXPHOS (Figure 4C and Figure 5B). In leukemia cells autophagy inhibition by ATG7 depletion resulted in a switch from glycolysis to OXPHOS to either compensate for the reduction in energy levels generated by glucose metabolism, or the elevated ATP production by increased OXPHOS impaired glycolysis [53]. However, we observed an increase in both glycolysis and OXPHOS. As AML cells heavily rely on OXPHOS for cell survival and were shown to be particularly sensitive to mitochondria damaging agents [73,74,75,76], we observed increased sensitivity of ATG3-deficient AML cells to OXPHOS inhibiting drugs oligomycin and antimycin A whereas ATG3-depleted AML cells were resistant glycolysis inhibition (Figure 5C). A recent study has demonstrated that AML cell lines differ in their energy metabolism. Suganuma et al. classify THP-1 cells as OXPHOS-dependent, based on the fact that the cells were more sensitive to oligomycin treatment than glycolysis inhibition [77]. However, we observed similar sensitivity of THP-1 cells to inhibition of OXPHOS and glycolysis, with increased sensitivity to OXPHOS inhibition in the absence of ATG3 (Figure 5C). This indicates that the dependency of AML cells shifts towards OXPHOS with deficient autophagy. Furthermore, targeting mitochondria is used as potential therapeutic target in combat against cancer [40,78,79] and the combination treatment of hydroxychloroquine together with mitoxantrone and etoposide (mitochondrial DNA inhibitors by topoisomerase II) is already in phase I clinical trials [76]. Besides that, Bhattacharya et al. reported the potential of using SBI-0206965, an autophagy inhibitor, combined with venetoclax in AML treatment [80]. In accordance with this, our data demonstrate that a combination of mitochondrial and autophagy inhibition may be a promising approach for treating leukemia. Considering that ATG3 has enzymatic activity, combining a specific inhibitor targeting ATG3 together with mitochondria targeting drugs may be a novel therapeutic option for AML treatment.

However, it remains unclear why AML cells upregulate glycolysis as well as OXPHOS upon autophagy inhibition and why the dependence on OXPHOS increases dramatically. Additionally, the molecular mechanism remains to be elucidated, including whether there is a direct relationship between the enzymatic function of ATG3 and the mitochondrial phenotype we observed. Moreover, our results obtained by cell culture experiments might differ from those obtained in AML patients, as the simplified cell culture conditions are different from the complexity of the human organism. Nevertheless, cancer cell lines are of great importance as a preclinical model system because, under the right conditions and with appropriate controls, they retain most of the genetic features of AML and further provide therapeutic insights [81]. To validate our findings, in vivo xenograft experiments would be required to further evaluate the potential of ATG3 inhibition as a therapeutic approach for the treatment of AML.

Taken together, our findings indicate the importance of ATG3 in the regulation of metabolism and survival of AML cells, as inhibition of mitochondrial oxidative phosphorylation severely impaired cell survival upon loss of ATG3. Moreover, targeting both autophagy and mitochondrial oxidative phosphorylation might be a potential therapeutic strategy for AML.

## Data Availability

Data are contained within the article and Supplementary Materials.

## Supplementary Materials

The following are available online at https://www.mdpi.com/article/10.3390/cancers13236142/s1, Supplementary Materials and Methods. File S1: Supplementary methods. Figure S1: CRISPR/Cas9 proliferation screen with autophagy library. Figure S2: Autophagy flux, LC3 lipidation, ATG7 and ATG4 expression, and autophagosome formation in ATG3-depleted AML cells. Figure S3: Colony formation, cell cycle, and apoptosis analysis upon loss of ATG3. Figure S4: Flow cytometry images of mitochondrial membrane potential, and mitochondrial mass, and western blot quantification of COXIV. Figure S5: Quantification of key parameters of mitochondrial function. Figure S6: Lactate production, ECAR measurements, and glutamine deprivation in ATG3-depleted AML cells. Figure S7: Full length membrane views of western blots. Table S1: Significant dropout genes from CRISPR/Cas9 autophagy screen in THP-1 and MV4-11 cells.
