# Supplementary material for: Metabolic Rewiring Is Essential for AML Cell Survival to Overcome Autophagy Inhibition by Loss of ATG3
Source: Cancers (Basel). 2021 Dec 6;13(23):6142. doi: 10.3390/cancers13236142 (PMC8657081; doi:10.3390/cancers13236142)
Supplement: Supplementary file 1 [file cancers-13-06142-s001.zip › cancers-1443280-supplementary.pdf]

Article

Metabolic rewiring is essential for AML cell survival to overcome autophagy inhibition by loss of ATG3

Fatima Baker [1,2], Ibrahim H. Polat [1], Khalil Abou-El-Ardat [1,2,3], Islam Alshamleh [2,4], Marlyn Thoelken [1], Daniel Hymon [2,4], Andrea Gubas [5], Sebastian E. Koschade [1,2,3,5], Jonas B. Vischedyk [1,3], Manuel Kaulich [3,5,6], Harald Schwalbe [2,4], Shabnam Shaid [1,7*], and Christian H. Brandts [1,2,3,7*]

[1] Department of Medicine II, Hematology/Oncology, Goethe University, 60590 Frankfurt am Main, Germany; baker@med.uni-frankfurt.de (F.B.); polat@med.uni-frankfurt.de (I.H.P.); khalilard@gmail.com (K.A-E-A.); thoelken@med.uni-frankfurt.de (M.T.); koschade@med.uni-frankfurt.de (S.E.K.); vischedyk@med.uni-frankfurt.de (J.B.V.)
[2] German Cancer Consortium (DKTK) and German Cancer Research Center (DKFZ), 69120 Heidelberg, Germany; alshamleh@nmr.uni-frankfurt.de (I.A.); hymon@nmr.uni-frankfurt.de (D.H.); schwalbe@nmr.uni-frankfurt.de (H.S.)
[3] Frankfurt Cancer Institute, 60596 Frankfurt am Main, Germany; kaulich@em.uni-frankfurt.de (M.K.)
[4] Center for Biomolecular Magnetic Resonance (BMRZ), Institute of Organic Chemistry and Chemical Biology, Goethe-University, 60438 Frankfurt am Main, Germany
[5] Institute of Biochemistry II, Faculty of Medicine, Goethe University, 60590 Frankfurt am Main, Germany; a.gubas@em.uni-frankfurt.de (A.G.)
[6] Cardio-Pulmonary Institute, 60590 Frankfurt am Main, Germany
[7] University Cancer Center Frankfurt (UCT), University Hospital, Goethe University, 60590 Frankfurt am Main, Germany
* Correspondence: shabnam.shaid@kgu.de (S.S.); christian.brandts@kgu.de (C.H.B.)

File S1. Supplementary Material and Methods

1.1. CRISPR/Cas9 proliferation screen

An autophagy library containing 876 guide RNAs against 192 genes including 4% non-targeting controls was used for the proliferation screen [18]. The sgRNA autophagy library was provided by the Frankfurt CRISPR/Cas Screening Center (FCSC), jointly operated by Dr. Manuel Kaulich and Dr. Ivan Dikic, Goethe University Frankfurt. For virus production the autophagy library was co-transfected with the packaging vectors pMD2.G and psPAX2 into HEK293T cells using 4.5 µg/mL Polyethylenimine (PEI) (Sigma-Aldrich, Taufkirchen, Germany). After 16 h of transfection medium was changed to DMEM supplemented with 5% FBS, and 1% penicillin/streptomycin and the supernatant containing produced lentivirus was collected 48 h afterwards. Cas9-expressing THP-1 and MV4-11 cells were transduced with serial virus dilutions and selected for 3 days with 2 µg/mL puromycin. The virus dilution leading to a MOI of ~0.3 was then chosen for subsequent experiments.

For transduction, THP-1 and MV4-11 cells expressing Cas9 were incubated with 5 µg/mL polybrene (Sigma-Aldrich, Taufkirchen, Germany, TR-1003) for 30 min before adding the virus. Then, the cells underwent spinfection for one hour at 34°C and 1000xg. 24 h later, medium of the cells was changed for fresh RPMI and transduced cells were selected with 2 µg/mL puromycin starting from 48 h after the transduction for the whole duration of the screen. Cells were taken and frozen for DNA isolation two days after transduction before starting puromycin selection as timepoint day 0 and again after 34 days counted from day 0 (See Figure 1A).

For genomic DNA isolation, cells were washed twice with 1x PBS and lysed overnight at 37°C in 1 mL TEX buffer (10 mM Tris-HCl pH 7.5, 1 mM EDTA pH 7.9, 0.5% SDS) containing 250 µg/mL proteinase K (Carl Roth, Karlsruhe, Germany) and 10 µg/mL RNase A (Sigma-Aldrich, Taufkirchen, Germany). For precipitation, 350 µL 5 M NaCl was added, vortexed, and

incubated for 30 min at 4°C. After centrifugation at 12.000xg for 30 min at 4°C the supernatant was taken and mixed with 2 mL ice cold 100% Ethanol, vortexed and incubated for 60 min at -80°C. Next, the samples were centrifuged at 5.000xg for 45 min at 4°C. Then, after discarding the supernatant, the pellet was washed using 2 mL 70% ice-cold ethanol and centrifuged at 12.000xg at 4°C for 30 min. The pellet was air dried, and DNA was dissolved in DNase-free water.

Sample preparation for sequencing was performed with two PCR reactions. Total amount of genomic DNA was calculated as follows: number of guides x coverage / MOI x 6.6 pg DNA. For the first PCR reaction (PCR1) 45 ng of plasmid DNA or 2 µg of genomic DNA per PCR reaction was used in a final volume of 50 µL, containing 25 µL Next High-Fidelity 2x PCR Master Mix (NEB, Frankfurt am Main, Germany), and 2.5 µL of each 10 µM PCR1 reverse and forward primers. Thermal cycler protocol was set as follows: Initial denaturation at 98°C for 2 min, 15 cycles of denaturation at 98°C for 2 min, annealing at 60°C for 55 sec, extension at 72°C for 1 min, and final extension at 72°C for 10 min. For the second PCR reaction (PCR2) 12.5 µL of PCR1 product was used in a final volume of 50 µL with 25 µL 2x Next High-Fidelity 2x PCR Master Mix, and 2.5 µL of 10 µM PCR2 primers containing Illumina adaptors and barcodes. Thermal cycler protocol was set as follows: Initial denaturation at 98°C for 3 sec, 15 cycles of denaturation at 98°C for 1 min, annealing at 68°C for 55 sec, extension at 72°C for 55 sec, and final extension at 72°C for 10 sec. PCR2 products were excised from a 2% agarose gel stained with SYBR Safe DNA gel stain (Invitrogen) and purified using Nucleospin Gel and PCR Clean-up kit (Macherey-Nagel, Dueren, Germany).

Genomic DNA sequencing experiments were performed with Illumina technology. Briefly, gel-purified PCR products of screening samples were denatured and diluted according to Illumina guidelines and set to a final concentration of 2.6 pM in a total volume of 2.2 mL and 15% PhiX control and loaded onto a MiSeq sequencer (Illumina, San Diego, CA, USA) aiming for a read counts allowing a 500- to 1,000-fold sequencing depth, according to the manufacturer's protocol. Sequencing was performed with paired end reads and 75 cycles, plus 8 cycles of index reading. Analysis was performed using MAGeCK with maximum likelihood estimation (version 0.5.9). Data of the different time points were normalized to d0.

1.1. Generation of stable ATG3 knockdown cells by lentiviral transduction

Knockdown of ATG3 was performed by lentiviral transduction using pLKO.1-puro vector with shRNA targeting human endogenous ATG3 (TRCN0000148120, Sigma-Aldrich, Taufkirchen, Germany, 5′-CCGGGATGTGACCATTGACCATATTCTCGAGAA-TATGGTCAATGGTCACATCTTTTTG-3′), ATG5 (TRCN0000330394, Sigma-Aldrich, Taufkirchen, Germany, 5′-CCGGCCTGAACAGAATCATCCTTAACTCGAGTTAAGGATGAT-TCTGTTCAGGTTTTTG-3′), and ATG7 (TRCN0000377305, Sigma-Aldrich, Taufkirchen, Germany, CCGGGGCGTGAGACACATCACATTTCTCGAGAAATGTGATGTGTCTCAC-GCCTTTTTG). Also, non-targeting shRNA (SHC002, Sigma-Aldrich, Taufkirchen, Germany, sequence: 5′-CCGGCAACAAGATGAAGAGCACCAACTC-3′) was used as control. For CRISPR/Cas9-mediated knockout cells, pKLV2.2-h7SKgRNA5(SapI)-hU6-gRNA5-PGKpuroBFP-W vector was used with gRNA targeting human *ATG3* (5′-GTAGATACAT-ATCACAACAC-3′), *ATG9A* (5′-TCAAGGCCGAGTACAAACGT-3′), *ATG12* (5′-CTCCCCAGAAACAACCACCC-3′), or non-human-targeting control (gNHT) (5′-AAC-TATTTCCTTTTTGTTTA-3′) at the hU6gRNA cassette. Lentivirus was produced transfecting HEK293T cells with shRNA-pLKO.1-puro and pKLV2.2-h7SKgRNA5(SapI)-hU6-gRNA5-PGKpuroBFP-W as described above.

For transduction, THP-1 and MV4-11 cells were incubated with 5 µg/mL polybrene (Sigma-Aldrich, Taufkirchen, Germany, TR-1003) for 30 min before adding lentiviruses in a 1:5 ratio and followed by spinfection for one hour at 34°C and 1000xg. Transduced cells were selected with 2 µg/mL puromycin for at least 3 days.

1.2. Cell growth analysis and colony formation assay

After transduction and 3 days of puromycin selection, expansion curves were performed. For cell expansion curves, $1x10^4$ cells were seeded in 100 µL growth medium and absolute cell numbers were determined by counting using Trypan Blue (Sigma-Aldrich, Taufkirchen, Germany) at day 2, 4, and 7. Fresh growth medium was added at each time point. For colony formation capacity determination, cells were seeded in medium containing methylcellulose MethoCult M3231 (STEMCELL Technologies, Saint Égrève, France) and colonies were counted microscopically at day 7 of culture.

1.3. Flow cytometry analyses

Flow cytometry analyses were performed in AML cell lines after 72 hours of puromycin selection. For measuring apoptosis, a staining with apoptotic marker APC-conjugated Annexin V (BD, 550475) and 7-AAD (BD, Heidelberg, Germany, 51-68981) was performed according to manufacturer's instructions. For cell cycle analysis APC BrdU Flow Kit (BD, Heidelberg, Germany, 552598) was used according to the manufacturer's instructions. To measure mitochondrial superoxide levels, cells were incubated with 5 µM MitoSOX Red Mitochondrial Superoxide Indicator (Thermo Fisher Scientific, Rockford, IL, USA, M36008) in culture medium for 10 min at 37°C in the dark. After washing with PBS, cells were measured by flow cytometry. For mitochondrial mass cells were stained with 20 nM MitoTracker Green FM (Cell Signaling, Danvers, MA, 9074) for 30 min at 37°C and measured by flow cytometry.

For measuring autophagy flux, $1x10^5$ GFP-rLC3B-RFP expressing cells were used. For glycolysis inhibition cells were incubated with 1 mM 2-Deoxy-D-glucose (2-DG) (Sigma-Aldrich, Taufkirchen, Germany) for 24 h. Mt-mKEIMA expressing cells were treated with 400 µM deferiprone (DFP) for 16 h or 10 µM oligomycin/antimycin A (O/A) for 8 h. Afterwards, GFP, RFP, or pH change of the cells were measured by flow cytometry.

1.4. Western Blotting

For total protein extraction cells were lysed as described previously [7] in NP40 lysis buffer supplemented with 9.2 mM sodium orthovanadate, and 0.1 mg/mL PMSF. Proteins were separated by 4-12% Bis-Tris polyacrylamide gel electrophoresis and transferred onto nitrocellulose membranes. Primary antibodies for ATG3 (Santa Cruz, sc-393660, 1:500), ATG7 (Santa Cruz, sc-376212, 1:500), ATG4 (Cell Signaling, Danvers, MA, 7613, 1:1000), LC3B (Novus Biologicals, NB100-2220, 1:1000), COXIV (Cell Signaling, Danvers, MA, 4844, 1:1000), and ß-Actin (Sigma-Aldrich, Taufkirchen, Germany, A2228, 1:10000) and corresponding secondary antibodies (goat anti-mouse IgG-HRP (Jackson ImmunoResearch, 115-030-003, 1:10000) and goat anti-rabbit IgG-HRP (Jackson ImmunoResearch, 111-036-047, 1:10000)) were used. Western blot quantification densitometry analysis was performed using ImageJ Software (NIH) normalizing all data to the loading control.

1.5. Immunofluorescence staining

To analyze autophagosome formation immune fluorescence staining was performed as described previously [7] after 72 hours of puromycin selected AML cells. Primary antibody mouse anti-LC3 (MBL Life science, M152-3, 1:50) and secondary antibody Alexa Fluor 594 goat anti-mouse (Thermo Fisher Scientific, Rockford, IL, USA, A-11005, 1:300) were used. Nuclei were stained using 1 µM SYTOX Green nucleic acid stain (Thermo Fisher Scientific, Rockford, IL, USA, S7020). For quantification of LC3 punctae ImageJ (v2.1.0, NIH, Bethesda, MD, USA) was used. At least 100 cells per replicate and condition of five z sections were analyzed for LC3 signal intensity per cell after subtraction of nuclear (SytoxGreen) signal and background.

1.6. Assessment of Mitochondrial and Glycolytic Activity

To assess mitochondrial and glycolytic activity AML cells were transduced with shRNA containing virus, selected for three days, and $1x10^4$ cells in 100 μL media were seeded in a 96 well plate and incubated at 37°C with 5% CO_2. After 72 hours, on one hand, cells were counted using Trypan Blue and on the other hand 25 μL CellTiter-Glo reagent was added to each well. Subsequently, the plate was incubated for 10 min and luminescence was measured using Tecan infinite M200 Pro plate reader. ATP/cell ratio was calculated by dividing luminescence signal to the cell number in the corresponding well.

After 72 hours of puromycin selection, $2x10^5$ cells were washed twice with basal Dulbecco's Modified Eagle Medium (Sigma-Aldrich, Taufkirchen, Germany, D5030) and transferred to a XF96 Polystyrene Cell Culture Microplate (Agilent, Santa Clara, CA, USA) in the corresponding medium and incubated at 37°C for one hour without CO_2 before measurement. For OCR Dulbecco's Modified Eagle Medium supplemented with 10 mM glucose, 2 mM glutamine, and 1 mM pyruvate was used and OCR was measured in the presence of 2 μM oligomycin, 2 μM carbonyl cyanide 4-(trifluoromethoxy) phenylhydrazone (FCCP), and antimycin and rotenone, each 2 μM, respectively, from the XF Cell Mito Stress Test Kit (Agilent, Santa Clara, CA, USA).

For ECAR measurements cells were washed and seeded in a XF96 Polystyrene Cell Culture Microplate (Agilent, Santa Clara, CA, USA) as described above. Measurement was performed in Dulbecco's Modified Eagle Medium supplemented with 2 mM glutamine in the presence of 10 mM glucose, 2 μM oligomycin, and 2 μM FCCP.

1.7. NMR spectroscopy

THP-1 and MV4-11 cells were transduced with shRNA against ATG3 or non-targeting control and selected for four days with 2 μg/mL puromycin. Cells were set at a density of $0.3x10^6$ cells/mL 24 h prior to harvesting. $1x10^7$ THP-1 and $1.5x10^7$ MV4-11 cells were harvested by centrifugation at 2000xg for 3 min at RT, washed with PBS and pelleted for 20 seconds at 13000xg at 4°C. Metabolite extraction was performed as described previously [19]. Cell extracts were dried at 4°C for 4 h in a vacuum dryer.

For unlabeled NMR measurements cells were resuspended in 190 μL NMR phosphate buffer (100 mM sodium phosphate in 100% D_2O (Deutero, Kastellaun, Germany) with 0.5 mM trimethylsilypropanoic acid (TMSP) (reference compound) (Sigma-Aldrich, Taufkirchen, Germany, pH 7)). Samples were sonicated afterwards for 10 min and loaded into NMR tubes. A Bruker AV600MHz spectrometer was used for measurements equipped with a nitrogen-cooled triple resonance probe head (5 mm TCI Prodigy, ^1H, ^{13}C, ^{15}N Z-GRD). Tuning, matching, and shimming were performed, and experiments were recorded using the NOESYGPPR1D pulse sequence implemented in the Bruker standard pulse sequence library at 25°C with 512 number of scans, 12 ppm spectral width (SW) and 32K TD. Spectral processing was performed in NMRLab [20]. Metabolites identification was performed using the Chenomx profiler software (Chenomx Inc. Edmonton, Alberta, Canada) as described previously [21]. Metabolites quantification was performed in NMRLab and intensities were normalized according to TMSP peak and a fold change was calculated relative to non-targeting control.

For the tracer-based experiment, cells were grown in glucose-free RPMI (Gibco, Thermo Fisher Scientific, Rockford, IL, USA) supplemented with 10% FCS, 2 mM glutamine, 1% penicillin/streptomycin, and 10 mM D-Glucose U-$^{13}C_6$ (Sigma-Aldrich, Taufkirchen, Germany) 24 h prior to harvest. $1x10^7$ THP-1 and $2.4x10^7$ MV4-11 cells were harvested after 24 h as described above. Extracts were resuspended in 40 μL NMR phosphate buffer, sonicated, and loaded into NMR tubes. A Bruker AV600MHz spectrometer with a nitrogen-cooled triple resonance probe head (1.7 mm TCI MicroCryoProbe) was used. Spectra were recorded using the pulse sequence HSQCCTPHPRSP implemented in the Bruker standard pulse sequence library at 25°C with 32 scans, 1428 points in the direct and 1468 points in the indirect dimension. Spectral processing

was performed in TopSpin 4.0.2. Metabolites identification and assignment was performed using NMRFAM-Sparky and the Human Metabolome Database.

1.8. Spectrophotometry assays

Glucose and glutamine consumption, and lactate production rates in cells were analyzed by measuring the decrease or increase in the concentration of extracellular glucose, glutamine, or lactate in the medium at 72 h, compared to their initial concentrations in the culture medium. They were normalized to the cell number at the beginning and end of the experiment. $1x10^5$ cells were seeded in 1 mL RPMI medium after transduction followed by 3 days of puromycin selection and incubated for 72 hours at 37°C with 5% CO_2. The extracellular glucose concentration at a given time point was measured by calculating the decrease in NAD(P)H concentration caused by the conversion of total glucose by hexokinase and conversion of resulting glucose-6-phosphate into D-gluconate-6-phosphate by G6PD. To measure glutamine concentration, glutamine was first converted to glutamate by glutaminase (GLS) reaction by incubating the media samples with 125 mU/mL glutaminase in 125 mM acetate buffer (pH 5) with soft agitation for 30 min at 37 °C. Then, glutamate concentration was determined by further conversion of glutamate to α-ketoglutarate by glutamate dehydrogenase (GDH) reaction and measurement of NADH change. Lactate concentration was determined by measuring the change in NADH concentration resulting from the lactate dehydrogenase (LDH) reaction, which was carried out at 37°C by adding medium to a 96 well plate containing 1.22 mg/mL NAD and 87.7 U/mL LDH in 0.2 M hydrazine 12 mM EDTA buffer, pH 9. All the measurements were done using a plate reader infinite 200 Pro (Tecan) and measuring the absorbance of NAD(P)H at 340 nm.

2. Supplementary Figures

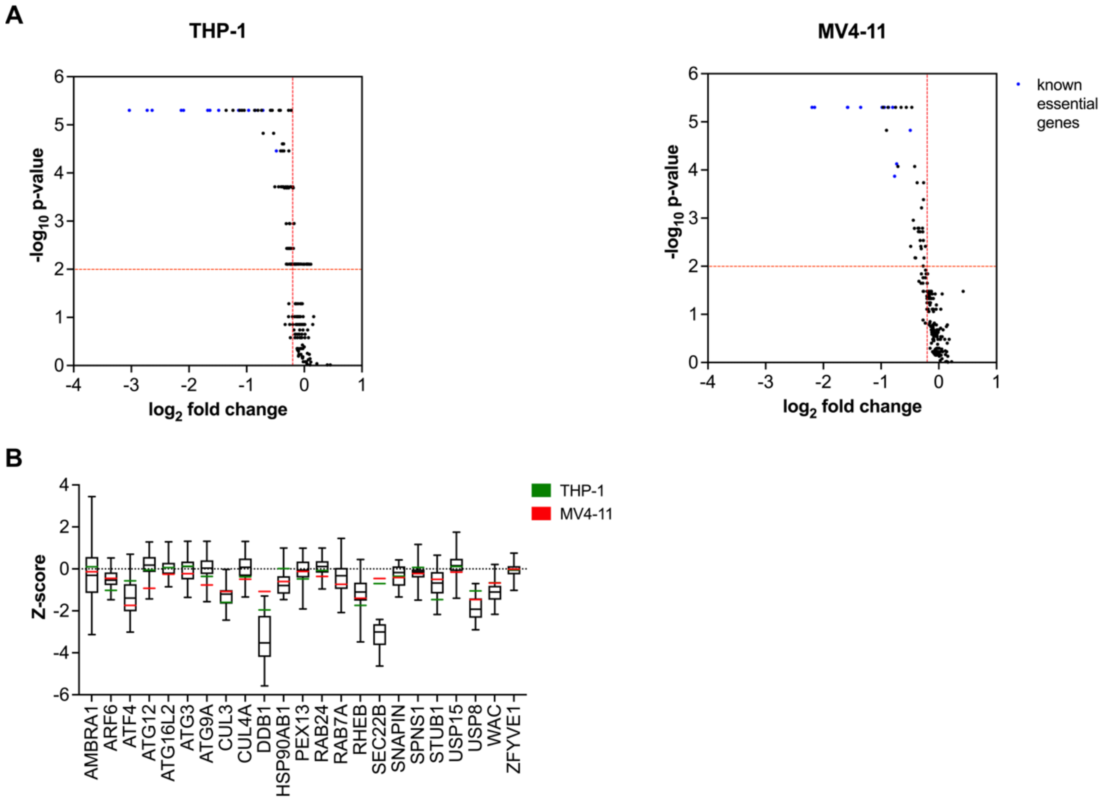

Figure S1. CRISPR/Cas9 proliferation screen with autophagy library. (**A**) Scatter plot of all genes including known essential genes from our CRISPR/Cas9 proliferation screen with the autophagy library in THP-1 and MV4-11 cells. (**B**) Box-Whiskers plot (min–max) with z-scores calculated for the 23 dropout hits in THP-1 and MV4-11 cells. Z-scores were compared to 40 CRISPR/Cas9 proliferation screens available over iCSDB (integrated database of CRISPR screens [23]).

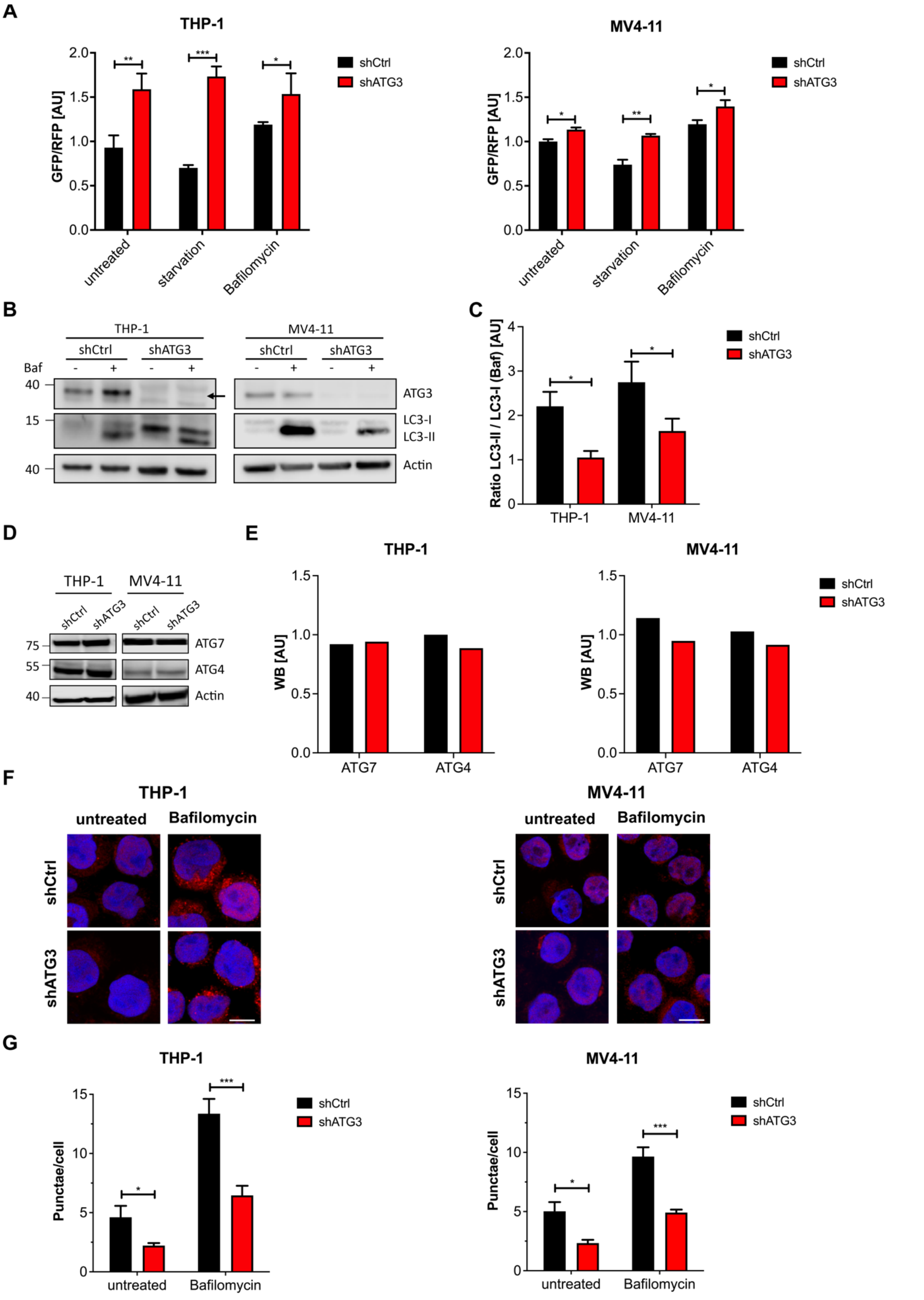

Figure S2. Autophagy flux, LC3 lipidation, ATG7 and ATG4 expression, and autophagosome formation in ATG3-depleted AML cells. (**A**) Autophagy flux was measured by the GFP-LC3B-RPF reporter in THP-1 and MV4-11 cells using flow cytometry. As controls autophagy induction was performed by incubating cells in PBS as starvation condition for 4 hours and for blockage of autophagy cells were treated with 100 nM Bafilomycin for 4 hours.

(**B**) Western blot of LC3-lipidation and (**C**) quantification of LC3 levels by LC3-II/LC3-I ratio. (**D**) Western blot of ATG7 and ATG4 expression and (**E**) quantification of expression levels normalized to actin levels. (**F**) Representative confocal microscopy images of immunostaining against endogenous LC3B. For inhibition of autophagosomal degradation 100 nM bafilomycin A1 was used for 4 hours. Scale bars measures 10 μM. (**G**) Quantification of LC3 punctae per cell using ImageJ. Student's t test was performed in (A, C and G). Error bars represent SEM. $^{*}p < 0.05$, $^{***}p < 0.001$.

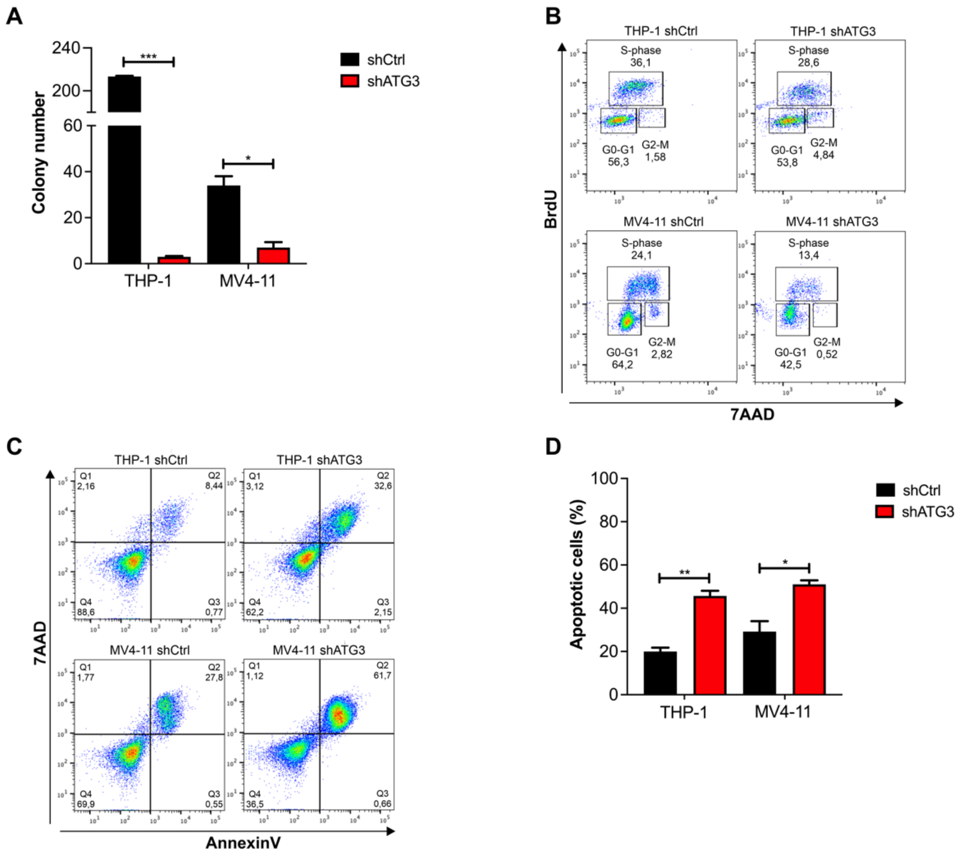

Figure S3. Colony formation, cell cycle, and apoptosis analysis upon loss of ATG3. (**A**) Colony formation assay of control or ATG3-depleted (shATG3) THP-1 and MV4-11 cells. Colonies were counted at d7. (**B**) Dot plots of THP-1 and MV4-11 control and ATG3-depleted cells stained with BrdU-7AAD for cell cycle analysis. Representative flow cytometry images are shown. (**C**) Dot plots of THP-1 and MV4-11 control and ATG3-depleted cells stained with annexin V/7AAD for apoptosis. Representative flow cytometry images are shown. Q2 plus Q3 represent apoptotic cells. (**D**) Quantification of apoptotic cells. Student's t test was performed in (A, D). Error bars represent SEM. *p < 0.05, **p < 0.01, ***p < 0.001.

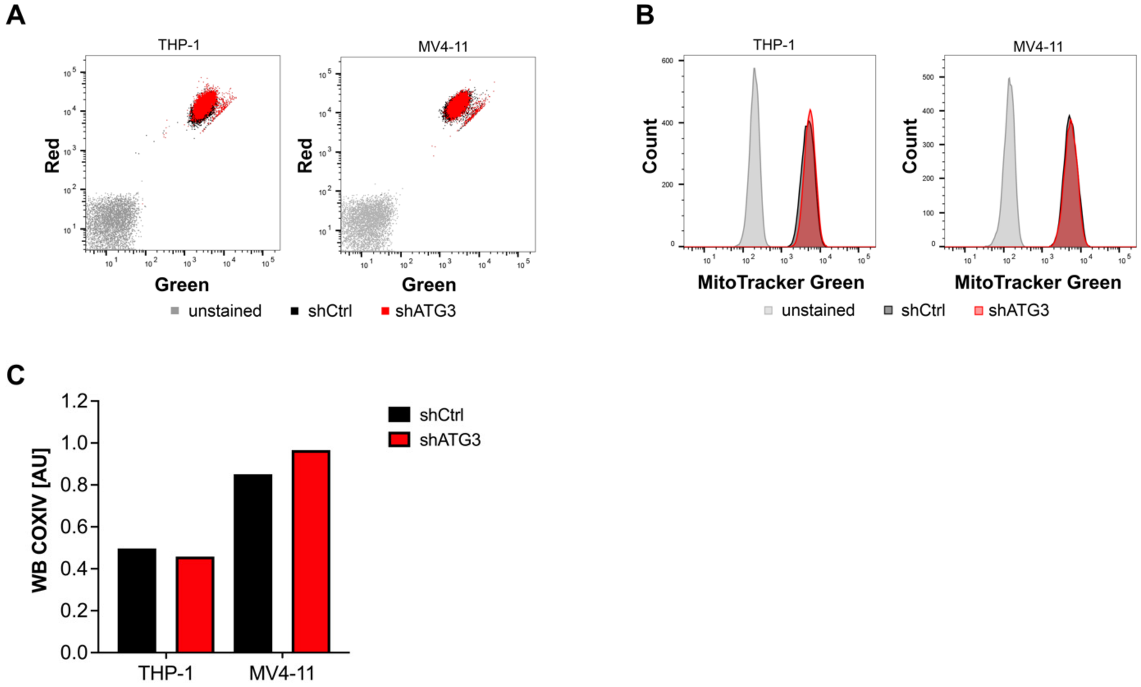

Figure S4. Flow cytometry images of mitochondrial membrane potential, and mitochondrial mass, and western blot quantification of COXIV. (**A**) Mitochondrial membrane potential was determined in THP-1 and MV4-11 cell lines by flow cytometry using JC-1 staining. A representative flow cytometry images is shown. (**B**) Mitochondrial mass was assessed by MitoTracker green staining and measured by flow cytometry. A representative image is shown. (**C**) For quantification COXIV antibody signal was normalized to actin signal.

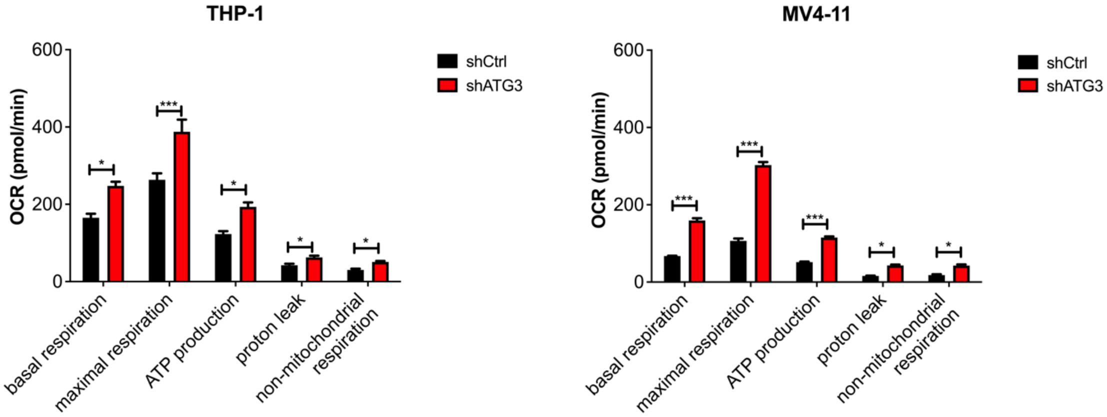

Figure S5. Quantification of key parameters of mitochondrial function. OCR measurements were performed using the Agilent Seahorse XFe96 Analyzer and key parameter were quantified according to manufacturer's instructions. Student's t-test was performed. Error bars represent SEM. ns, not significant, *$p < 0.05$, **$p < 0.01$, ***$p < 0.001$.

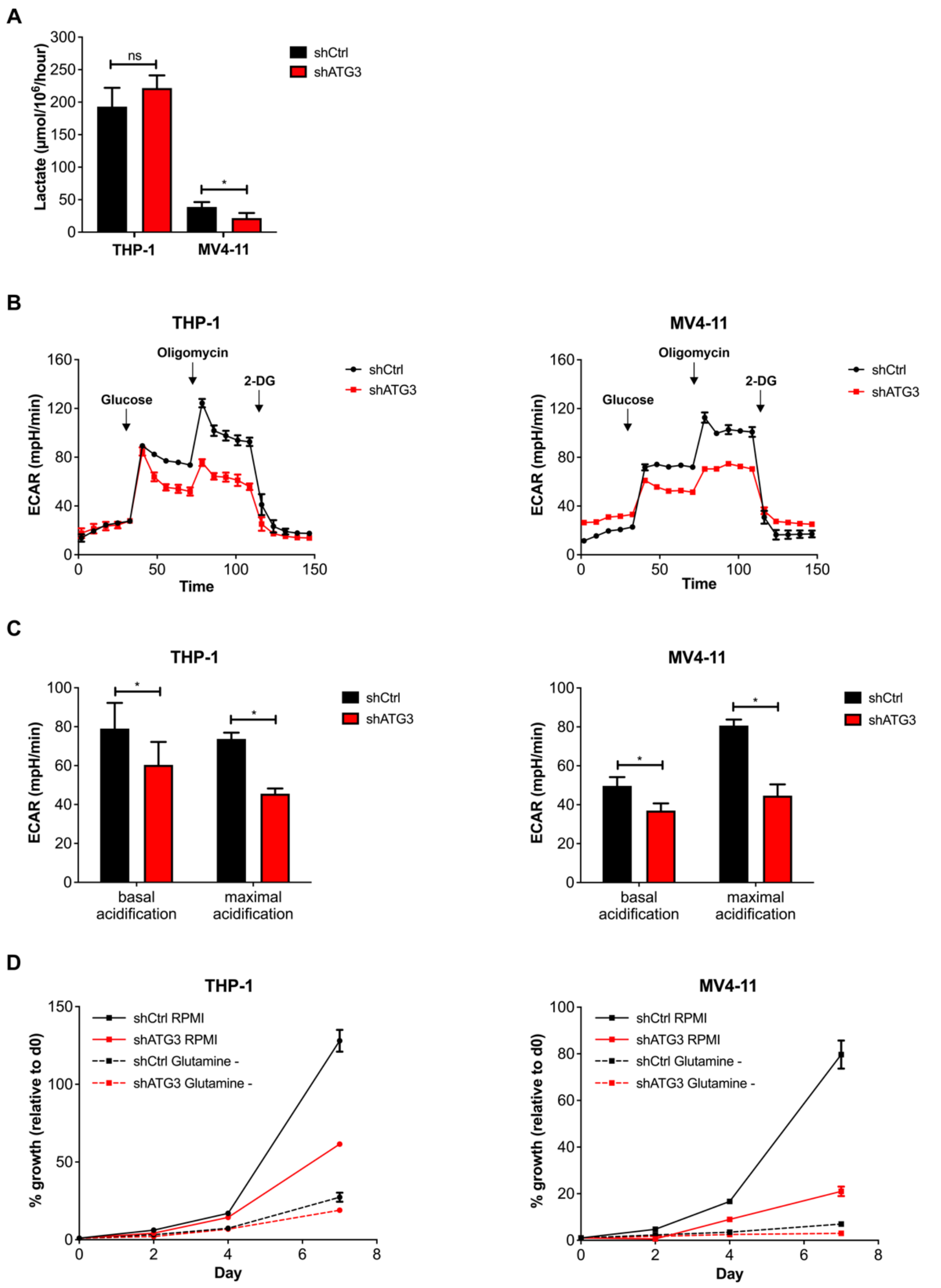

Figure S6. Lactate production, ECAR measurements, and glutamine deprivation in ATG3-depleted AML cells. (**A**) Extracellular lactate concentration was measured by spectrophotometric assay after incubating control and ATG3-depleted THP-1 and MV4-11 cells in normal RPMI medium for 72 h. (**B**) Lactate excretion was analyzed in control and ATG3-depleted cells by extracellular acidification rate (ECAR) measured in real time by the Agilent Seahorse XFe96 Analyzer. Representative experiments are shown. (**C**) Quantification of ECAR. Basal acidification shows the difference of basal ECAR and after glucose injection. Maximal acidification shows the difference after oligomycin injection compared to glucose injection. (**D**) Growth analysis of THP-1 and MV4-11 cells with normal RPMI medium compared to medium without glutamine. 10.000 cells were seeded on day 0 and counted on indicated days with Trypan blue exclusion. Student's t-test was performed in (A, C). Error bars represent SEM. ns, not significant, $^*p < 0.05$.

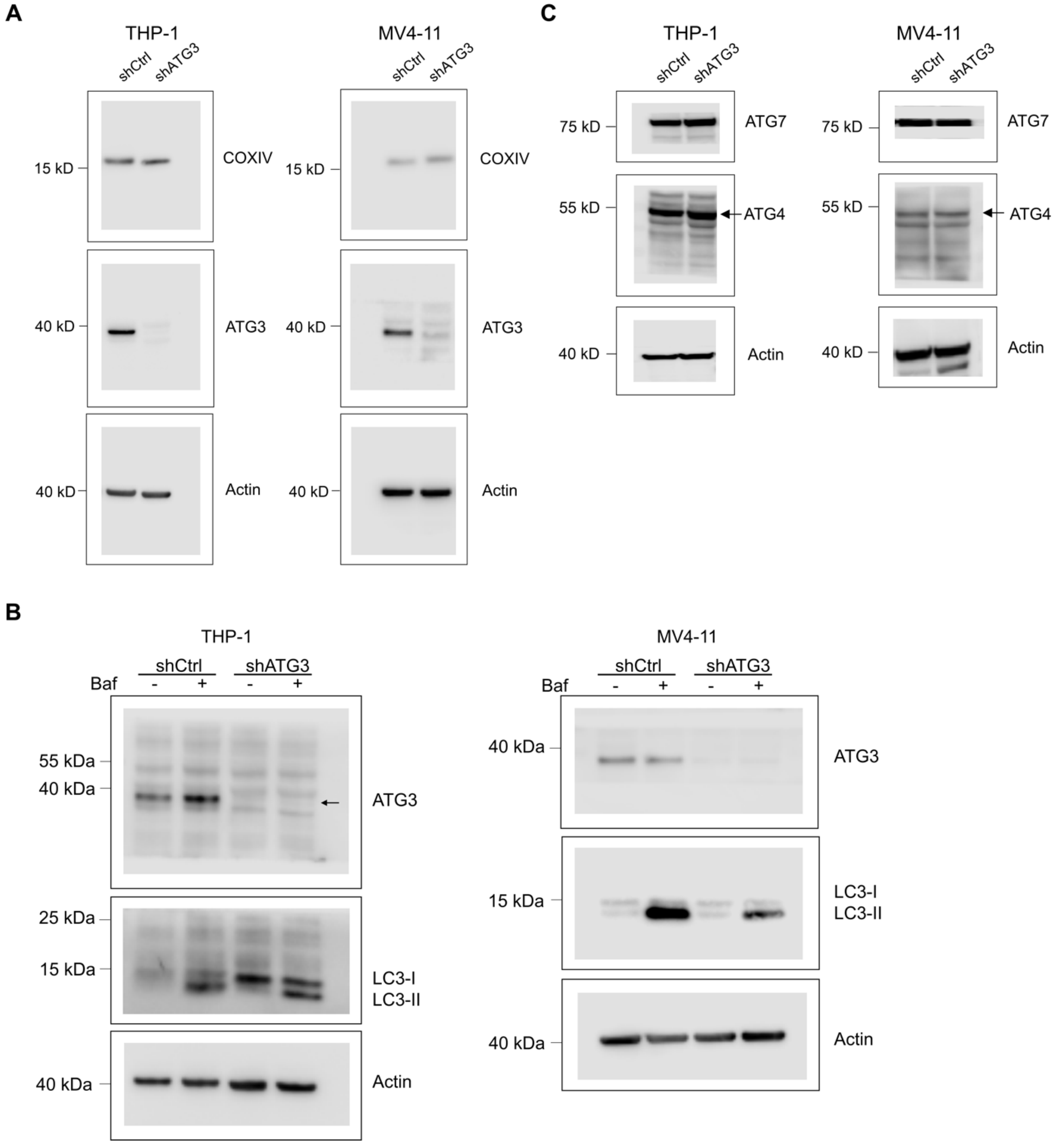

Figure S7. Full length membrane views of western blots. (**A**) Uncropped western blot images of Figure 2E. (**B**) Uncropped western blot image of Figure S2B. (**C**) Uncropped western blot images of Figure S2D.

3. Supplementary Tables

Table S1. Significant dropout genes from CRISPR/Cas9 autophagy screen in THP-1 and MV4-11 cells. Significance threshold was set to $-\log_{10}$ p-value ≥ 2 and dropout threshold to $\log_2$ fold change ≤ -0.2.

**Cell line**	**THP-1**	**MV4-11**	**Known essential genes for cell proliferation**
**Number of dropout genes**	**74**	**44**	**13**
**Gene names**	AMBRA1	AMBRA1	BCL2
	ARF6	ARF6	CDK1
	ATF4	ATF4	MAPK1
	ATG12	ATG101	MTOR
	ATG16L2	ATG12	NDC80
	ATG2A	ATG16L2	PHB2
	ATG3	ATG3	PIK3C3
	ATG4C	ATG9A	PLK1
	ATG4D	BCL2	RPL11
	ATG9A	CDK1	RPL5
	ATG9B	CUL3	RPS19
	BAG1	CUL4A	RPS7
	BCL2	DDB1	VCP
	BNIP3	E2F1	
	CAMKK2	GABARAP	
	CDK1	HSP90AB1	
	CREB1	KEAP1	
	CUL3	LAMP1	
	CUL4A	MAPK1	
	DDB1	MTOR	
	DRAM2	NDC80	
	EPG5	PEX13	
	HDAC6	PHB2	
	HSP90AB1	PIK3C3	
	HSPA8	PLK1	
	LGALS3	RAB24	
	MAP1LC3A	RAB7A	
	MAPK1	RHEB	
	MTOR	RPL11	
	NDC80	RPL5	
	NEDD4	RPS19	
	NEDD4L	RPS7	
	OPTN	SEC22B	
	PARK7	SNAPIN	

	PEX13	SPNS1	
	PEX3	STUB1	
	PHB2	TMEM41B	
	PI4K2A	ULK1	
	PIK3C3	USP15	
	PINK1	USP36	
	PLK1	USP8	
	PRKAA1	VCP	
	RAB1A	WAC	
	RAB24	ZFYVE1	
	RAB7A		
	RB1CC1		
	RGS19		
	RHEB		
	RPL11		
	RPL5		
	RPS19		
	RPS7		
	RUBCN		
	SAR1A		
	SEC22B		
	SEC62		
	SNAPIN		
	SPNS1		
	SREBF2		
	STUB1		
	STX8		
	TBC1D5		
	TECPR2		
	TFEB		
	TRIM21		
	USP10		
	USP15		
	USP8		
	UVRAG		
	VAMP8		
	VCP		
	WAC		
	WDR45B		
	ZFYVE1		
